# In Vivo Ca_V_3 Channel Inhibition Promotes Maturation of Glucose-Dependent Ca^2+^ Signaling in Human iPSC-Islets

**DOI:** 10.3390/biomedicines11030807

**Published:** 2023-03-07

**Authors:** Kaixuan Zhao, Yue Shi, Jia Yu, Lina Yu, Martin Köhler, Amber Mael, Anthony Kolton, Thomas Joyce, Jon Odorico, Per-Olof Berggren, Shao-Nian Yang

**Affiliations:** 1 The Rolf Luft Research Center for Diabetes and Endocrinology, Karolinska Institutet, SE-171 76 Stockholm, Sweden; 2 Regenerative Medical Solutions, Inc., Madison, WI 53719, USA

**Keywords:** anterior chamber of the eye (ACE), calcium channel, cytoplasmic-free Ca^2+^ concentration ([Ca^2+^]_i_), stem cell, in vivo confocal microscopy, islet

## Abstract

Ca_V_3 channels are ontogenetically downregulated with the maturation of certain electrically excitable cells, including pancreatic β cells. Abnormally exaggerated Ca_V_3 channels drive the dedifferentiation of mature β cells. This led us to question whether excessive Ca_V_3 channels, retained mistakenly in engineered human-induced pluripotent stem cell-derived islet (hiPSC-islet) cells, act as an obstacle to hiPSC-islet maturation. We addressed this question by using the anterior chamber of the eye (ACE) of immunodeficient mice as a site for recapitulation of in vivo hiPSC-islet maturation in combination with intravitreal drug infusion, intravital microimaging, measurements of cytoplasmic-free Ca^2+^ concentration ([Ca^2+^]_i_) and patch clamp analysis. We observed that the ACE is well suited for recapitulation, observation and intervention of hiPSC-islet maturation. Intriguingly, intraocular hiPSC-islet grafts, retrieved intact following intravitreal infusion of the Ca_V_3 channel blocker NNC55-0396, exhibited decreased basal [Ca^2+^]_i_ levels and increased glucose-stimulated [Ca^2+^]_i_ responses. Insulin-expressing cells of these islet grafts indeed expressed the NNC55-0396 target Ca_V_3 channels. Intraocular hiPSC-islets underwent satisfactory engraftment, vascularization and light scattering without being influenced by the intravitreally infused NNC55-0396. These data demonstrate that inhibiting Ca_V_3 channels facilitates the maturation of glucose-activated Ca^2+^ signaling in hiPSC-islets, supporting the notion that excessive Ca_V_3 channels as a developmental error impede the maturation of engineered hiPSC-islet insulin-expressing cells.

## 1. Introduction

Human-induced pluripotent stem cells (hiPSCs) have successfully been used to produce spherical aggregates of islet hormone-producing cells, referred to here as hiPSC-islets, through in vitro cultivation [1,2,3,4,5,6]. However, clinical hiPSC-islet transplantation as a curative treatment for diabetes still remains challenging because hiPSC-islets cannot fully mature in vitro into glucose-responsive insulin-secreting islets and may reliably achieve functional maturation post-transplantation [1,3,6,7,8,9,10,11]. Transplanted hiPSC-islets can gradually gain the ability to release detectable insulin in response to elevated blood glucose. Nevertheless, they are not competent enough to tightly control glucose homeostasis, largely due to inappropriate glucose-activated Ca^2+^ signaling [1,3,6]. They have yet to fully gain this indispensable signaling pathway for accurate glucose-stimulated insulin secretion (GSIS) at different blood glucose levels [1,3,6]. As is well known, glucose-induced increases in cytoplasmic-free Ca^2+^ concentrations ([Ca^2+^]_i_) trigger and orchestrate accurate GSIS, which has widely been deemed as the gold standard for determining the maturity and clinical applicability of surrogate islets, including hiPSC-islets [1,3,6,12,13,14,15,16,17,18]. Therefore, measurements of [Ca^2+^]_i_ dynamics are of utmost importance in understanding the functional maturation of hiPSC-islets. However, it has not been possible to retrieve single, intact hiPSC-islet grafts, and thus, [Ca^2+^]_i_ dynamics could not be directly measured in these grafts without losing their in vivo gained phenotypes. This inspired us to exploit the anterior chamber of the eye (ACE) of immunodeficient mice, which has been used as a reliable transplantation site for hiPSCs and their derivative [5,19,20,21,22]. The ACE is filled with aqueous humor endowed with high oxygen and less stress and may serve as a unique niche for the in vivo maturation of single, non-aggregated hiPSC-islet grafts. These grafts can then be treated with locally applied substances and retrieved intact for direct ex vivo [Ca^2+^]_i_ measurements. Herein, we demonstrate the ability to precisely assess [Ca^2+^]_i_ dynamics induced by glucose across a wide range of concentrations in hiPSC-islet grafts.

It is well known that the short in vitro development of engineered hiPSC-islets induced by a limited number of factors gives rise to a series of developmental errors in contrast to the long ontogenesis of native islets in the maternal and infant body [1,3,6]. This prompted us to hypothesize that the short in vitro development of hiPSC-islets does not fully mimic the long in vivo ontogenesis of native human islets, resulting in overexpression or hyperactivation of Ca_V_3 channels as a brake on the maturation of glucose-activated Ca^2+^ signaling in hiPSC-islet grafts. We came up with this hypothesis based on the following facts. Ca_V_3 channels are appreciably expressed in a diverse range of immature cells and even embryonic stem cells but are only preserved to a certain extent in some mature, electrically excitable cells, including pancreatic β cells [23,24,25]. Correspondingly, these channels mediate Ca^2+^ influx into embryonic stem cells to ensure their self-renewal capacity [24,25] but are downregulated and conduct a relatively small proportion of Ca^2+^ currents in mature human islet β cells, while they cannot be detected in mature mouse islet β cells [12,13,15,18,26]. Low-voltage-activated Ca_V_3 channels are strikingly different from high-voltage-activated Ca^2+^ (Ca_V_) channels, which mediate Ca^2+^ influx only at stimulatory blood glucose levels, as they competently activate various Ca^2+^ signaling pathways at both fasting and stimulatory blood glucose levels [12,13,15,18]. This enables Ca_V_3 channels to persistently participate in the preservation of β cell maturity by mediating a mild, continuous Ca^2+^ influx and consequently controlling Ca^2+^-dependent gene expression in β cells [12,13,15,18,24]. Conversely, in certain pathological contexts, such as when Ca_V_3 channels undergo elevated expression, they mediate exaggerated Ca^2+^ influx, thereby exhausting β cell maturity-related Ca^2+^-dependent gene expression machinery, and/or activate pathological Ca^2+^ signaling detrimental to β cell maturity [12,13,15,18,27,28,29,30]. We have tested our hypothesis by performing direct ex vivo [Ca^2+^]_i_ measurements in single intraocular hiPSC-islet grafts retrieved intact following intravitreal infusion of the Ca_V_3 channel blocker NNC55-0396. Our data reveal that intravitreal infusion of NNC55-0396 gives rise to reduced basal [Ca^2+^]_i_ levels and increased glucose-stimulated [Ca^2+^]_i_ responses in hiPSC-islets, demonstrating that downregulation of Ca_V_3 channel activity can correct the erroneous in vitro development of hiPSC-islets to promote the in vivo maturation of glucose-dependent Ca^2+^ signaling in these engineered islets.

## 2. Materials and Methods

### 2.1. In Vitro Cultivation and Differentiation of HiPSCs

The procedure has been described elsewhere [5,19]. In brief, the undifferentiated hiPSC line NCRM-1 (Lonza/NIH, Walkersville, MD, USA) was cultured in mTeSR1 medium (Stem Cell Technologies, Vancouver, BC, Canada) on Growth Factor Reduced Matrigel (Corning, Corning, NY, USA) for 3–4 days at 37 °C in a humidified 5% CO_2_ incubator. When cells became almost confluent, they were subjected to single-cell dispersion, seeded at 5 × 10^5^ cells/mL into Matrigel-coated Transwell culture plates (Corning) containing mTeSR1 medium (Stem Cell Technologies) and cultured for 24 h. Then, their differentiation was induced sequentially under six different medium conditions. At the end of the six-stage induction, cells were removed from the Transwell culture plates, resized and placed into Ultra Low Attachment flasks (Corning) in suspension as stage seven for eight days. Upon completion of stage seven, the resulting hiPSC-islets were obtained for in vitro quality testing and intraocular transplantation [5,19]. All reagents were purchased from R&D Systems (Minneapolis, MN, USA) unless otherwise stated.

### 2.2. Animals

NOD-scid IL2Rgamma^null^ (NSG) mice aged from 8 to 10 weeks were obtained from Charles River Laboratories (Sulzfeld, Germany). They were kept in temperature- and humidity-controlled rooms under standard 12 h light/12 h dark conditions and had unlimited access to food pellets and tap water all day. Mice were selected as human islet transplant recipients at random. All animal experiments were approved by the Regional Ethical Committee at Karolinska Institutet (Dnr 2-4532_2022, approved 15 December 2022).

### 2.3. ACE Transplantation

Transplantation of hiPSC-islets into the ACEs of immunodeficient mice was performed as previously described [5,19]. In brief, recipient mice were subjected to general anesthesia with a mixture of 2.5% isoflurane and 40% oxygen via a nose mask, and their head and eyeball were immobilized with a head holder and an eyeball holder, respectively. Then, hiPSC-islets were carefully sucked into a donor tissue-delivering glass micropipette connected by tygon tubing to a threaded plunger syringe. Thereafter, a tiny corneal incision was made by cautiously puncturing the cornea with an insulin syringe needle (29 G). The tip of the micropipette preloaded with hiPSC-islets was prudently inserted into the ACE through the corneal incision. The preloaded hiPSC-islets were gently extruded into the ACE. Lastly, the tip of the micropipette was slowly removed from the corneal incision. At this step, extra care should be exercised to avoid escape of hiPSC-islets from the hyperbaric ACE [5,19]. After ACE transplantation, the recipient mice were detached from the head and eyeball holders and kept lying on their side before anesthesia recovery. To relieve surgical pain, recipients were treated with the analgesic drug buprenorphine (0.1 mg/kg/day, s.c.) after ACE transplantation [31]. During that time and thereafter, the recipient mice behaved similar to non-operated ones, with no evidence suggestive of pain, poor vision or blindness, as documented in our previous studies [31,32].

### 2.4. Intravitreal Infusion

Mice transplanted with hiPSC-islets were anesthetized and immobilized as described above. Here, 30 µM NNC55-0396 (Tocris, Bristol, UK) or vehicle solution was sucked into a beveled glass micropipette with a tip diameter of about 20 µm, which was connected to a 100-microliter microsyringe (Hamilton, Reno, NV, USA) and mounted on a micromanipulator. Thereafter, the sclera of the eyeball containing hiPSC-islets was penetrated by the tip of an insulin syringe needle (29 G) to obtain a tiny scleral hole. Then, immediately, the NNC55-0396 solution-containing micropipette was gently placed into the vitreous body through the scleral hole under an upright stereomicroscope (Leica Microsystems Heidelberg GmbH, Mannheim, Germany). Subsequently, 1.5 µL NNC55-0396 or vehicle solution was slowly extruded by 3 intermittent infusions during 60 min into the vitreous cavity with a UNIVENTOR 802 syringe pump (Univentor, Zejtun, Malta). Intravitreal infusion was performed once a day for a week.

### 2.5. In Vivo Confocal Microscopy

Anesthetized and immobilized recipient mice were laid under an upright Leica DM6000 CFS microscope fitted with a Leica TCS SP5 II confocal laser scanner (Leica Microsystems). Their eyeball was positioned at an angle suitable for microscopic imaging by carefully adjusting the head and eyeball holders. Engrafted hiPSC-islets of interest were visualized using a long-distance water-dipping lens (HXC IRAPO L25 ×/0.95 W) dipped into Viscotears on the cornea. The backscattering signal was detected at 630–640 nm from hiPSC-islet grafts illuminated with a 633 nm laser light. Vascularization of hiPSC-islet grafts was imaged following the injection of 0.4 µmol/L Qtracker 565 (Thermo Fisher Scientific, Waltham, MA, USA) in 100 µL saline into the tail vein of recipient mice. The fluorescence emitted from the intravascular Qtracker 565 excited by a 488 nm laser line was acquired at 550–585 nm. Z-stack images were collected every 2–3 µm and processed with LAS AF Software (version: 2.7.7.12402, Leica Microsystems) and Volocity (version: 6.3.0, PerkinElmer, Waltham, MA, USA). The relative intensity of intraocular hiPSC-islet backscatter was derived by normalizing the mean voxel intensity of intraocular islet backscatter to that of iris backscatter. The vascular density of intraocular islets was represented by the percentage of vascular volume in intraocular islet volume obtained from stack imaging of intraocular islet backscatter. Mouse body temperature was kept at 37 °C for the duration of image acquisition [5,19].

### 2.6. Intraocular HiPSC-Islet Graft Retrieval

Recipient mice were anesthetized, and their heads and eyeballs were immobilized as described above at 1–2 days after 1-week intravitreal infusion. Intraocular hiPSC-islet graft retrieval was conducted under a stereomicroscope. To begin with, the entire cornea and iris engrafted with hiPSC-islets were removed from recipient mice by cutting along the border between the cornea and the sclera, i.e., the corneal limbus, with a pair of curved eye scissors. Subsequently, the iris together with hiPSC-islet grafts was peeled off from the cornea. Single, intact hiPSC-islet grafts with a tiny piece of the iris at their periphery were dissected out. The obtained single hiPSC-islet grafts were used for ex vivo [Ca^2+^]_i_ measurements or patch clamp recordings.

### 2.7. Ex Vivo [Ca^2+^]_i_ Measurements

Fura-2 loading was performed in retrieved intraocular hiPSC-islet grafts. The grafts were incubated with 2 µM fura-2 LeakRes/AM for 60 min at 37 °C in HEPES-buffered solution containing 125 mM NaCl, 5.9 mM KCl, 2.56 mM CaCl_2_, 1.2 mM MgCl_2_, 25 mM HEPES, 2 mM glucose and 0.1% bovine serum albumin (pH 7.4). Thereafter, fura-2-loaded hiPSC-islet grafts were placed and immobilized onto a glass coverslip at the bottom of a recording chamber. [Ca^2+^]_i_ was measured using a Spex Fluorolog spectrophotometer coupled to a Zeiss Axiovert 35 M microscope with a Zeiss Fluar 40×/l.30 oil objective (Carl Zeiss, Göttingen, Germany). The fura-2 F340/F380 ratio was registered to denote [Ca^2+^]_i_ [33]. During a recording, a hiPSC-islet graft was continuously perifused with HEPES-buffered solution supplemented with 2 mM glucose, 20 mM glucose or 30 mM KCl at 37 °C. Evtra software was employed to analyze the obtained data [27].

### 2.8. Cultivation of Dispersed Cells of Intraocular HiPSC-Islet Grafts

HiPSC-islet grafts were retrieved from the recipient ACE and dispersed in Ca^2+^-free medium with accutase (Gibco, Carlsbad, CA, USA). Dispersed hiPSC-islet cells were seeded into Petri dishes; fed CMRL 1066 culture medium (ICN Biomedicals, Santa Ana, CA, USA) supplemented with HEPES (10 mM), L-glutamine (2 mM), gentamycin (50 mg/mL), fungizone (0.25 mg/mL), ciprofloxacin (20 mg/mL), nicotinamide (10 mM) and 10% fetal bovine serum and maintained at 37 °C in a humidified 5% CO_2_ incubator [34].

### 2.9. Patch Clamp Recordings

Conventional whole-cell currents were recorded from cultured hiPSC-islet cells. Recording electrodes were made from borosilicate glass capillaries and fire-polished. Their resistance was between 4 and 6 MΩ when electrodes were filled with intracellular solution and bathed in extracellular solution. The intracellular solution consisted of 110 mM CsCl, 30 mM CsF, 10 mM EGTA, 10 mM HEPES and 4 mM MgATP (pH adjusted with CsOH to 7.2). The extracellular solution contained 140 mM NaCl, 9 mM BaCl_2_, 1 mM CaCl_2_, 10 mM HEPES and 2 mM glucose (pH adjusted with NaOH to 7.4). Whole-cell current recordings were taken from cells held at −80 mV, depolarized to +20 mV for 5 ms and repolarized to −70 mV with an EPC-9 patch clamp amplifier at room temperature (about 22 °C). Pulse/PulseFit (version: 8.80, HEKA Elektronik, Lambrecht/Pfalz, Germany) and IGOR program (version: 6.0.1.0, WaveMetrics, Portland, OR, USA) were employed to acquire and analyze whole-cell currents. The amplitude of the whole-cell currents was normalized to cell capacitance. The β cell identity was verified by insulin mRNA positivity [35].

### 2.10. Single-Cell RT-PCR

The entire hiPSC-islet cell was collected into a PCR tube with 10 µL 1 × Colorless GoTaq^®^ reaction buffer containing MgCl_2_ (Promega, Madison, WI, USA) with a glass micropipette immediately after a whole-cell current recording and stored at −70 °C for later use. Insulin mRNA in collected cells was detected using the QIAGEN OneStep RT-PCR Kit (Valencia, CA, USA) according to the manufacturer’s instructions. The RNasin Plus RNase Inhibitor (Promega) was added to protect mRNAs. The insulin primer pair including the forward primer 5′ AACGAGGCTTCTTCTACACACC 3′ and the reverse primer 5′ TTCCACAATGCCACGCTTCTG 3′ was synthesized by Sigma-Aldrich (St. Louis, MO, USA). The final concentration of the primers in the reaction mix was 0.6 µM. PCR amplification was carried out for 30 cycles of 94 °C for 1 min, 60 °C for 1 min and 72 °C for 1 min. The obtained PCR products were detected by 2% agarose gel electrophoresis and SYBR™ Gold Nucleic Acid Gel Stain (Thermo Fisher Scientific) [35].

### 2.11. Data Analysis

Data are presented as mean ± SEM overlaid with individual points. Statistical significance was determined by one-way ANOVA followed by the least significant difference (LSD) test or Student’s *t*-test. The significance level was set to 0.05.

## 3. Results

### 3.1. Ex Vivo Measurements of Glucose-Activated [Ca^2+^]_i_ Dynamics in Single, Intact HiPSC-Islet Grafts Retrieved from the ACE following In Vivo Local Treatment

**Figure 1:**
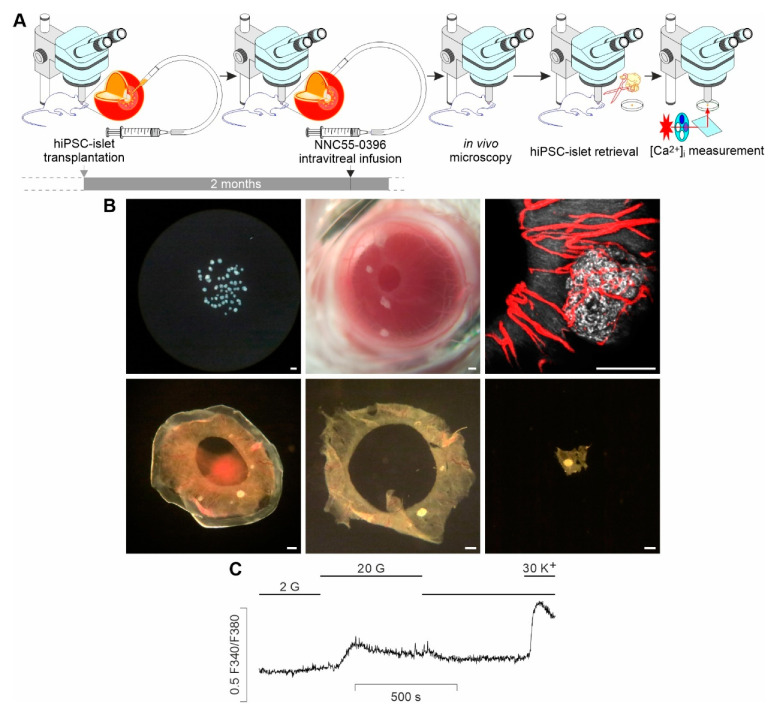
Transplantation of single, non-aggregated hiPSC-islets onto the immunodeficient mouse iris, intravitreal infusion of NNC55-0396, in vivo microscopy, intact retrieval of single intraocular grafts and ex vivo [Ca^2+^]_i_ measurements in retrieved single intraocular grafts. (**A**) Experimental design schematic. (**B**) Stereomicroscopic graphs and confocal microscopic images showing hiPSC-islets of suitable size (<250 µm in diameter) selected for ACE transplantation (**upper left**); single, non-aggregated hiPSC-islets engrafted onto the iris (**upper middle**); light backscatter (grey) merged with fluorescence of Qtracker 565 (red) injected into the lateral tail vein (**upper right**) from an intraocular hiPSC-islet graft; single, non-aggregated hiPSC-islet grafts sandwiched between the iris and cornea excised as a whole from the eyeball (**lower left**); single, non-aggregated hiPSC-islet grafts engrafted on the iris peeled from iris–cornea sandwich (**lower middle**) and an intact hiPSC-islet graft attached to a small piece of the iris (**lower right**). Scale bar = 200 µm. (**C**) A typical [Ca^2+^]_i_ trace illustrating glucose- and K^+^-induced [Ca^2+^]_i_ responses registered from a single hiPSC-islet graft retrieved at 2 months post-transplantation following intravitreal infusion of NNC55-0396.

To make ex vivo assessments of glucose-activated [Ca^2+^]_i_ dynamics in single, intact hiPSC-islet grafts retrieved from the ACE following in vivo local treatment possible, we combined ACE transplantation, intravitreal infusion, intact hiPSC-islet graft harvest and ex vivo [Ca^2+^]_i_ measurements (Figure 1A).

Initially, we transplanted 5–10 hiPSC-islets into immunodeficient mouse ACEs and scattered them in the pupillary zone of the iris by gently extruding them through a glass micropipette connected to a syringe. The properly scattered hiPSC-islets adhered or attached to the iris following transplantation (Figure 1A,B). This dispersed layout of single, non-aggregated hiPSC-islets in the desired area is a prerequisite for the intact retrieval of single, non-aggregated and intact hiPSC-islet grafts from the recipient ACE. During the post-transplantation period, hiPSC-islets were progressively engrafted and vascularized on the iris (Figure 1B, upper middle and right). Their gross morphology, engraftment, vascularization and light scattering signals were monitored by using non-invasive in vivo stereomicroscopy/confocal microscopy (Figure 1A,B, upper middle and right). A representative stereomicrograph displays hiPSC-islets engrafted on the iris at 60 days post-transplantation (dpt) (Figure 1B, upper middle). A sample confocal micrograph shows a typically vascularized hiPSC-islet graft giving sufficient light scattering signals at 57 dpt (Figure 1B, upper right).

Within a week before the end of two months post-transplantation, infusion of NNC55-0396 was applied through a glass micropipette penetrating into the vitreous space through the sclera of recipient mice (Figure 1A). Caution should be exercised with regards to the total volume of infused solution, the flow rate of infusion and the position for penetration of glass micropipette. A total volume of 1.5 µL NNC55-0396 or vehicle solution was intermittently infused into the vitreous body for 60 min in the present study.

Following the intravitreal infusion, vascularization and light scattering signals of intraocular hiPSC-islet grafts were imaged and quantified by using noninvasive in vivo confocal microscopy (Figure 1A,B, upper right). Thereafter, retrieval of intact single, non-aggregated hiPSC-islet grafts from the iris of recipient mice was performed (Figure 1A,B, lower). During this procedure, the key is to avoid mechanical damage to hiPSC-islet grafts. Therefore, single, non-aggregated hiPSC-islet grafts sandwiched between the iris and cornea were carefully removed from the eyeball of the recipient mice (Figure 1B, lower left). Then, the hiPSC-islet grafts of interest were prudently excised by gently peeling the iris from the cornea and surgically incising the iris at the peripheral area of individual hiPSC-islet grafts with the greatest care under a stereomicroscope (Figure 1B, lower middle and right). Subsequently, excised hiPSC-islet grafts were subjected to routine [Ca^2+^]_i_ measurements (Figure 1A,C). Figure 1C shows a typical [Ca^2+^]_i_ trace illustrating glucose- and K^+^-induced [Ca^2+^]_i_ responses registered from a hiPSC-islet graft retrieved 2 months post-transplantation following intravitreal infusion of NNC55-0396.

### 3.2. Intravitreal Infusion of NNC55-0396 Promotes Glucose-Activated Ca^2+^ Signaling in Intact HiPSC-Islet Grafts Harvested from the ACE

**Figure 2:**
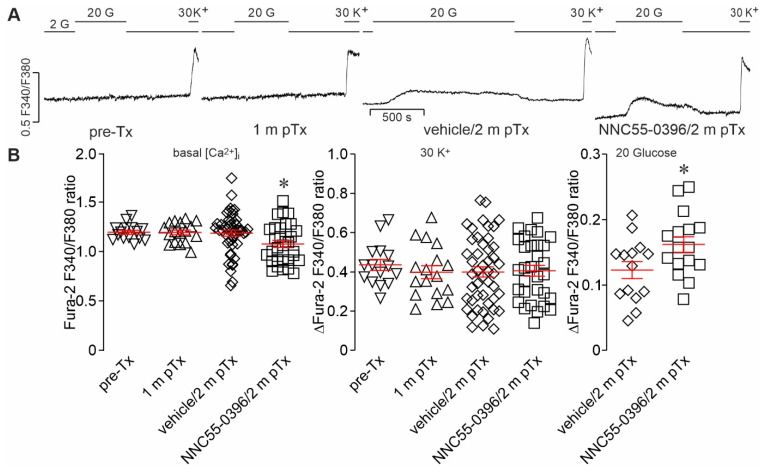
Effects of intravitreal infusion of NNC55-0396 on [Ca^2+^]_i_ dynamics in intraocular hiPSC-islet grafts retrieved intact. (**A**) Sample traces of fura-2 F340/F380 ratios acquired during sequential perifusion of 2 mM glucose, 20 mM glucose or 30 mM KCl in pre-transplanted hiPSC-islets (pre-Tx); intact, single intraocular hiPSC-islet grafts retrieved l month post-transplantation (1 m pTx) or 2 months post-transplantation following intravitreal infusion of vehicle (vehicle/2 m pTx) or NNC55-0396 (NNC55-0396/2 m pTx). (**B**) Quantification of average fura-2 F340/F380 ratios in pre-Tx, 1 m pTx and 2 m pTx during exposure to 2 mM glucose (n = 15 for pre-Tx group, n = 17 for 1 m pTx, n = 47 for vehicle/2 m pTx group, n = 31 for NNC55-0396/2 m pTx group) (**left**) and stimulation with 30 mM KCl (n = 15 for pre-Tx group, n = 17 for l m pTx group, n = 47 for vehicle/2 m pTx group, n = 31 for NNC55-0396/2 m pTx group) (**middle**) or 20 mM glucose (n = 14 for vehicle/2 m pTx group, n = 16 for NNC55-0396/2 m pTx group) (**right**). * *p* < 0.05.

The feasibility and merits of ex vivo [Ca^2+^]_i_ measurements in single intact hiPSC-islet grafts directly retrieved from the ACE enabled us to clarify whether inhibition of Ca_V_3 channel activity promotes the in vivo development of glucose-activated Ca^2+^ signaling in hiPSC-islets. Ex vivo [Ca^2+^]_i_ measurements showed that either pre-transplanted hiPSC-islets or hiPSC-islet grafts retrieved from the ACE at one month post-transplantation displayed K^+^ depolarization-induced [Ca^2+^]_i_ responses but were unresponsive to glucose stimulation (Figure 2A,B, middle). When hiPSC-islet grafts were maintained in recipient mice for two months, however, retrieved grafts exposed to either NNC55-0396 or vehicle solution displayed [Ca^2+^]_i_ responses to both elevated glucose and K^+^ depolarization (Figure 2A,B, middle and right), indicating progressive maturation between 1 and 2 months in the ACE. Importantly, the NNC55-0396-treated hiPSC-islet grafts showed increased [Ca^2+^]_i_ responses to glucose stimulation in comparison to vehicle solution-treated hiPSC-islet grafts and lower basal [Ca^2+^]_i_ than vehicle solution-treated hiPSC-islet grafts as well as pre-transplanted hiPSC-islets and intraocular hiPSC-islet grafts retrieved at one month post-transplantation (Figure 2A,B, left and right). There were no significant differences in basal [Ca^2+^]_i_ between the latter three groups (Figure 2B, left). Together, these data indicate that the inhibition of Ca_V_3 channels promotes glucose-activated Ca^2+^ signaling in intraocular hiPSC-islet grafts.

### 3.3. Ca_V_3 Currents Are Present in Insulin-Expressing Cells of Intraocular HiPSC-Islet Grafts and Remain Unaltered after Intravitreal Infusion of NNC55-0396

**Figure 3:**
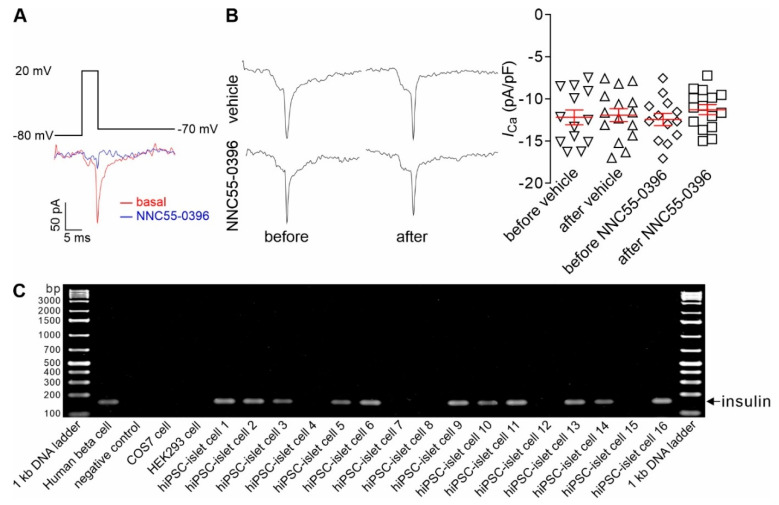
Effects of intravitreal infusion of NNC55-0396 on Ca_V_3 currents in insulin-expressing cells of retrieved intraocular hiPSC-islet grafts. (**A**) Slow deactivating tail current traces evoked by a depolarizing voltage pulse from −80 mV to +20 mV for 5 ms back to −70 mV (**upper panel**) in an insulin-positive cell of intraocular hiPSC-islet grafts retrieved at 2 months post-transplantation before (red) and when exposed to NNC55-0396 during patch clamp recordings (blue). (**B**) Sample slow deactivating tail current traces (**left**) and quantification of the average density of slow deactivating tail currents (**right**) registered in insulin-positive cells of intraocular hiPSC-islet grafts retrieved at 2 months post-transplantation following intravitreal infusion of vehicle or NNC55-0396; n = 13 for group before intravitreal infusion of vehicle (before vehicle), n = 15 for group after intravitreal infusion of vehicle (after vehicle), n = 13 for group before intravitreal infusion of NNC55-0396 (before NNC55-0396) and n = 15 for group after intravitreal infusion of NNC55-0396 (after NNC55-0396). (**C**) RT-PCR analysis of cDNA generated by reverse transcription of mRNA in insulin-positive cells of intraocular hiPSC-islet grafts retrieved after patch clamp recordings as well as a human islet β cell, negative control (sterile ultrapure water), COS7 cell and HEK293 cell with specific primers for insulin (146 bp amplicon).

The above observed phenomenon that [Ca^2+^]_i_ responds to both glucose and K^+^ stimulation in hiPSC-islet grafts indicates that Ca_V_ channels, including Ca_V_3 channels, may operate in insulin-expressing cells of intraocular hiPSC-islet grafts. To verify this possibility, we measured Ca_V_3 currents in these cells by recording slow deactivating tail currents after intravitreal infusion of vehicle or NNC55-0396. The identity of individual cells subjected to patch clamp recordings was corroborated by insulin mRNA positivity. A standard RT-PCR protocol using human insulin-specific primers revealed the expected 146 bp amplicon for insulin in hiPSC-islet cells (Figure 3C). As shown in Figure 3A,C, a cell from an hiPSC-islet graft exhibited clear Ca_V_3 currents, which were effectively blocked by acute application of NNC55-0396. Importantly, this cell was insulin mRNA-positive (hiPSC-islet cell 6). A sample of agarose gel electrophoresis analysis shows that a 146 bp cDNA fragment of human insulin was detected in single hiPSC-islet cells and native human islet cells as a positive control, but not in the negative control (sterile ultrapure water), COS7 and HEK 293 cells (Figure 3C). Furthermore, we also examined if sustained alteration in Ca_V_3 channels occurred in insulin-expressing cells of intraocular hiPSC-islet grafts retrieved from the ACE previously infused with NNC55-0396 for a week. In the absence of NNC55-0396, Ca_V_3 current density did not significantly differ between the two groups either before or after the pre-treatments (Figure 3B). These data verify that functional Ca_V_3 channels are present in insulin-expressing cells of hiPSC-islet grafts as the target of NNC55-0396 but did not alter their density and intrinsic functionality after 1-week intravitreal infusion of NNC55-0396.

### 3.4. Intravitreal Infusion of NNC55-0396 Has No Influence on Vascularization and Light Backscattering of Intraocular HiPSC-Islet Grafts

There is a possibility that the promoted glucose-activated Ca^2+^ signaling in intraocular hiPSC-islet grafts following the inhibition of Ca_V_3 channels may occur secondary to the improved vascularization, engraftment and survival of these islet grafts induced by the intravitreal infusion of NNC55-0396. Therefore, we investigated whether hiPSC-islet grafts exposed to NNC55-0396 differed from vehicle solution-treated ones in vascularization and light backscattering.

**Figure 4:**
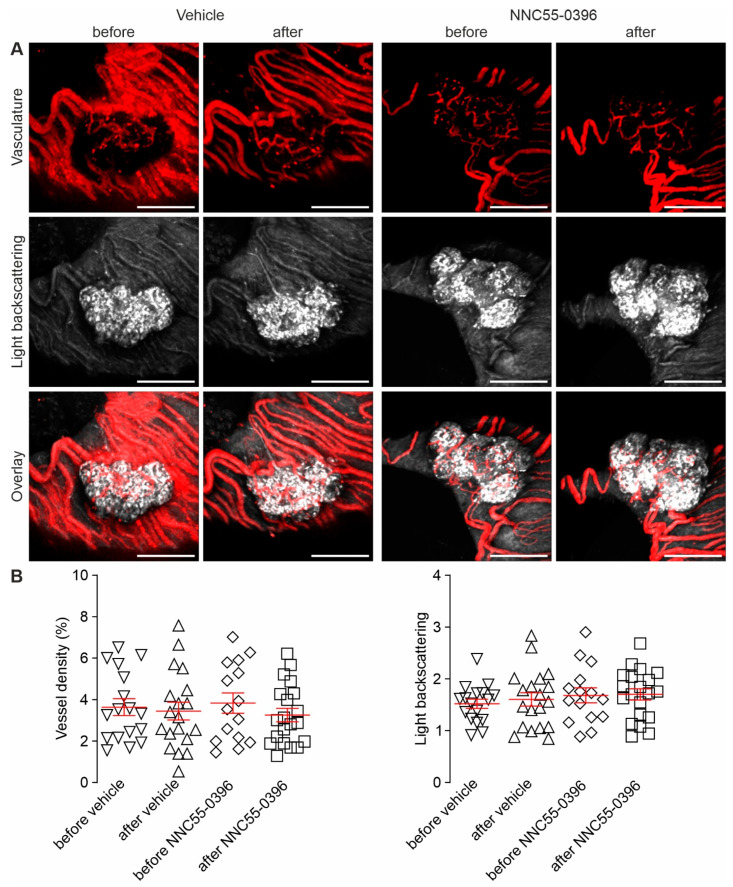
Effects of intravitreal infusion of NNC55-0396 on vascularization of and light backscatter from intraocular hiPSC-islet grafts. (**A**) Representative in vivo confocal images of the Qtracker 565-labelled vasculatures (**upper**) in and backscattered light (**middle**) from a single intraocular hiPSC-islet graft and their overlays (**lower**). Scale bar = 200 µm. (**B**) Quantification of vessel density (n = 17 for group before vehicle, n = 19 for group after vehicle, n = 15 for group before NNC55-0396, n = 20 for group after NNC55-0396) and light backscattering intensity (n = 17 for group before vehicle, n = 19 for group after vehicle, n = 15 for group before NNC55-0396, n = 20 for group after NNC55-0396) in single intraocular hiPSC-islet grafts before and after intravitreal infusion of vehicle or NNC55-0396.

By taking advantage of the ACE technology established by combining the transplantation of islets into the ACE and intravital microimaging of them non-invasively and longitudinally, we monitored the vascularization of single hiPSC-islet grafts before and after intravitreal infusion of NNC55-0396. In vivo confocal microscopy demonstrated that Qtracker 565 injected into the tail vein of recipient mice quickly appeared in vasculatures in hiPSC-islet grafts with and without exposure to NNC55-0396 (Figure 4A, upper and lower). Appreciable microvascular networks were distributed throughout a single hiPSC-islet graft prior to and following the administration of NNC55-0396 or vehicle solution (Figure 4A, upper and lower). Vascular density quantification detected no significant differences between NNC55-0396- and vehicle-exposed groups either before or after the exposures (Figure 4B, left). It is clear that intravitreal infusion of NNC55-0396 did not interfere with the vascularization of hiPSC-islet grafts.

Meanwhile, we also conducted non-invasive observation of light scattering signals, in parallel with that of vascularization in intraocular hiPSC-islet grafts exposed to NNC55-0396 or vehicle solution. As shown in the sample confocal images, hiPSC-islet grafts emitted similar light scatter signals prior to intravitreal infusion of NNC55-0396 or vehicle solution (Figure 4A, middle and lower). Following these treatments, there was no appreciable alteration in their light scatter signals. The compiled data showed that the NNC55-0396-treated group did not significantly differ from the vehicle solution-treated group in the intensity of light scatter signals before and after the treatments (Figure 4B, right). For neither the NNC55-0396-treated group nor the vehicle solution-treated group did the intensity of the light scatter signals change following treatment (Figure 4B, right). These data verify that intravitreally infused NNC55-0396 or vehicle solution did not acutely influence the light scattering signals of intraocular hiPSC-islet grafts.

## 4. Discussion

As is well known, native pancreatic islets exist as many individual micro-organs. Within each of them, different types of endocrine cells are highly integrated into a basic morphological and functional unit to execute various glucose-dependent activities such as glucose-activated [Ca^2+^]_i_ signaling and GSIS [12,15,36,37,38]. Such a micro-organotypic architecture also arises within a pluripotent stem cell-derived islet (PSC-islet), reflecting the similar morphological development of PSC-islets [6]. However, a detailed analysis of glucose-activated [Ca^2+^]_i_ signaling responsible for GSIS in hiPSC-islet grafts with an intact micro-organotypic architecture has not been carried out. This is because transplanted hiPSC-islets are aggregated at routine transplantation sites and cannot be retrieved intact from there [1,3,6,39,40,41,42]. This practical dilemma has considerably hindered research on glucose-dependent [Ca^2+^]_i_ dynamics during in vivo development of hiPSC-islet grafts. Indeed, glucose-dependent [Ca^2+^]_i_ dynamics have been examined in insulin-expressing cells isolated from PSC-islet grafts by using single-cell dispersion followed by in vitro cultivation and in pre-transplanted PSC-islets [1,3,6]. Obviously, single-cell dispersion and in vitro cultivation unavoidably damage the cytoarchitecture of PSC-islet grafts and probably compromise the in vivo gained phenotypes of these surrogate islets. In this context, intravital imaging of [Ca^2+^]_i_ in islets transplanted into the ACE can be used as a valuable tool [43]. In the present work, we were able to retrieve single hiPSC-islet grafts that suffered no mechanical and chemical stress and keep their architecture and functionality intact after removal. They could be directly used for detailed analysis of [Ca^2+^]_i_ dynamics driven by a change in glucose concentration or other secretagogues or inhibitors.

During normal in vivo development, β cell Ca_V_3 channels ontogenetically change under the strict control of complex developmental clocks that result from the integration of genetically encoded programs with intrinsic and extrinsic cues [15,24,25,26,44,45,46,47]. This ontogenetic event is species-dependent [12,13,15,24,25,26]. A substantial number of functional Ca_V_3 channels exist as early as in embryonic stem cells to participate in the regulation of proliferation and self-renewal of these cells [24,25]. Developmentally downregulated Ca_V_3 channels make only a minor contribution to total Ca_V_ currents and Ca^2+^ influx and play a negligible role in high-glucose-induced insulin secretion when human β cells become fully mature [12,13,15,18,26]. However, it appears that a limited number of Ca_V_3 channels in human β cells, activated at both fasting and stimulatory blood glucose levels, mediate optimally fine-tuned and persistent Ca^2+^ influx for the promotion and preservation of Ca^2+^-dependent β cell gene expression and maturation [12,13,15,18]. By contrast, pathologically excessive Ca^2+^ influx mediated by exaggerated β cell Ca_V_3 channels drives β cell maturity dissipation, manifested as reduced expression of exocytotic proteins and impaired GSIS [28,29,30]. The present work shows that intraocular hiPSC-islet grafts following in vivo exposure for 2 months demonstrate glucose-dependent [Ca^2+^]_i_ dynamics, which has been deemed as one of the most essential events representing the functional maturation of β cells [6,12,13,14,15,16,17,18]. Importantly, it also reveals that intravitreal infusion of the Ca_V_3 channel blocker NNC55-0396 significantly facilitates the development of glucose-dependent [Ca^2+^]_i_ dynamics in intraocular hiPSC-islet grafts, as evidenced by lower basal [Ca^2+^]_i_ levels and stronger glucose-induced [Ca^2+^]_i_ responses. Furthermore, insulin-positive cells in hiPSC-islet grafts express functional Ca_V_3 channels, which also exist innately at the earliest stages of ontogeny [24,25]. These findings verify that the functional maturation of in vitro generated hiPSC-islets relies on both the exposure to complex in vivo environments and the downregulation of excessive Ca_V_3 channels. This suggests that short in vitro cultivation and the artificially created developmental cues that may exist in vitro cannot fully recapitulate the protracted in vivo development of human β cells, which results in abnormal exaggeration of β cell Ca_V_3 channels. This may impede the development of full glucose-dependent Ca^2+^ signaling in hiPSC-islet grafts. This also emphasizes the importance of the optimally fine-tuned and persistent Ca^2+^ influx mediated by a defined number of β cell Ca_V_3 channels in human β cell maturation.

For their survival and development, intraocular hiPSC-islet grafts need to be vascularized to receive nutrients, oxygen and humoral factors as well as dispose of metabolic wastes. The present work shows that intraocular hiPSC-islet grafts are indeed satisfactorily vascularized and such vascularization is not acutely influenced by intravitreal infusion of the Ca_V_3 channel blocker NNC55-0396. This substantiates that the immunodeficient mouse ACE allows satisfactory hiPSC-islet vascularization, which is a prerequisite for their survival, engraftment and development, thus being suitable for fostering the functional maturation of hiPSC-islets, including the development of glucose-activated Ca^2+^ signaling. Importantly, these data indicate that the Ca_V_3 channel blocker NNC55-0396 promotes the development of glucose-activated Ca^2+^ signaling by acting on Ca_V_3 channels in hiPSC-islet cells, not by improving graft vascularization. Concurrent with the vascularization, appreciable light scattering signals were emitted from intraocular hiPSC-islet grafts and remained unchanged after exposure to NNC55-0396. The alteration of glucose-dependent Ca^2+^ signaling induced by NNC55-0396 and the absence of changes in light scattering suggest that the maturation of insulin-secretory granules happens earlier than that of glucose-activated Ca^2+^ signaling, since light scattering signals reflect the abundance of insulin secretory granules [5,42].

## 5. Conclusions

The present work verifies that the immunodeficient mouse ACE can serve as a unique site not only for in vivo maturation of hiPSC-islet grafts but also for local applications of substances to manipulate and study such maturation. These grafts can be micro-imaged intravitally, non-invasively and longitudinally and also retrieved without suffering physical and chemical disturbances for more precise ex vivo studies, as exemplified here by [Ca^2+^]_i_ measurements. This offers a resource for the mechanistic evaluation of the ontogenetic development of human stem cell-derived islets or other organoids. Importantly, the present work also demonstrates that inhibiting Ca_V_3 channels facilitates the induction of glucose-dependent Ca^2+^ signaling in hiPSC-islets, supporting the notion that excessive Ca_V_3 channels as a development error operate in hiPSC-islet cells to impede the maturation of these cells. The findings point out that Ca_V_3 channel blockers may act as an accelerator for the functional maturation of hiPSC-islets. Furthermore, taken together with previously documented findings, those reported in the present work suggest that Ca_V_3 channel blockers could help treat disorders resulting from cell dedifferentiation, such as certain cancers and diabetes [16,18,48,49,50].

## Data Availability

All the data, associated protocols and materials for this study are available within the paper.
